# CRISPR/Cas9-Mediated Multiplex Genome Editing of JAGGED Gene in *Brassica napus* L.

**DOI:** 10.3390/biom9110725

**Published:** 2019-11-12

**Authors:** Qamar U Zaman, Wen Chu, Mengyu Hao, Yuqin Shi, Mengdan Sun, Shi-Fei Sang, Desheng Mei, Hongtao Cheng, Jia Liu, Chao Li, Qiong Hu

**Affiliations:** 1 Oil Crops Research Institute of Chinese Academy of Agricultural Sciences, Key Laboratory for Biology and Genetic Improvement of Oil Crops, Ministry of Agriculture and Rural Affairs, No. 2 Xudong 2nd Road, Wuhan 430062, China; qamaruzamanch@gmail.com (Q.U.Z.); 18040598479@163.com (W.C.); haomengyu@caas.cn (M.H.); 82101182187@caas.cn (Y.S.); s1264673467@163.com (M.S.); 15652142445@163.com (S.-F.S.); meidesheng@caas.cn (D.M.); chenghongtao@caas.cn (H.C.); liujia02@caas.cn (J.L.); 2 Graduate School of Chinese Academy of Agricultural Sciences, Beijing 100081, China

**Keywords:** JAGGED, CRISPR/Cas9, replum, lateral organs, BnA08-JAGGED-Like-NUBBIN, pseudoseeds, pod shattering resistance, *Brassica napus*

## Abstract

Pod shattering resistance is an essential component to achieving a high yield, which is a substantial objective in polyploid rapeseed cultivation. Previous studies have suggested that the *Arabidopsis* JAGGED (JAG) gene is a key factor implicated in the regulatory web of dehiscence fruit. However, its role in controlling pod shattering resistance in oilseed rape is still unknown. In this study, multiplex genome editing was carried out by the CRISPR/Cas9 system on five homoeologs (BnJAG.A02, BnJAG.C02, BnJAG.C06, BnJAG.A07, and BnJAG.A08) of the JAG gene. Knockout mutagenesis of all homoeologs drastically affected the development of the lateral organs in organizing pod shape and size. The cylindrical body of the pod comprised a number of undifferentiated cells like a callus, without distinctive valves, replum, septum, and valve margins. Pseudoseeds were produced, which were divided into two halves with an incomplete layer of cells (probably septum) that separated the undifferentiated cells. These mutants were not capable of generating any productive seeds for further generations. However, one mutant line was identified in which only a BnJAG.A08-NUB-Like paralog of the JAG gene was mutated. Knockout mutagenesis in BnJAG.A08-NUB gene caused significant changes in the pod dehiscence zone. The replum region of the mutant was increased to a great extent, resulting in enlarged cell size, bumpy fruit, and reduced length compared with the wild type. A higher replum–valve joint area may have increased the resistance to pod shattering by ~2-fold in JAG mutants compared with wild type. Our results offer a basis for understanding variations in *Brassica napus* fruit by mutating JAG genes and providing a way forward for other Brassicaceae species.

## 1. Introduction

Rapeseed (*Brassica napus*) belongs to the Brassicaceae (Cruciferae) family and possibly arose from interspecific hybridization between turnip rape (2n = 20, AA genome) and cabbage (2n = 18, CC genome) [1] approximately 7500 years ago [2]. Rapeseed is grown for the production of green fuel, human consumption, animal feed, and industrial purposes [3]. Rapeseed yield has always been a major concern over the breeding history of the crop, but conventional plant breeding mostly depends on the generation of new genetic variations based on phenotypically evaluated individuals and populations. Molecular biology and genetic engineering have created new approaches for breeders to overcome the main limitations of genotype selection, excluding the phenotypic basis [4]. Efforts have been made to breed plants with increased potential against biotic and abiotic stresses that contribute to yield losses of about 40% in the case of heat stress [5]. Among all other negatively contributing factors to yield, pod shattering causes 20% yield losses in the normal harvesting season and damages crop production up to 50% under severe climatic conditions [6]. The genetic diversity of pod shattering resistance in *B. napus* is very limited; this trait diversity was probably lost during the natural evolution of the species [7]. Pod shattering is a key factor affecting the mechanical harvesting of rapeseed. Mechanized rapeseed harvesting reduces labor costs and can improve rapeseed production [8], thereby ensuring the global oil supply.

Fully mature pods of *B. napus* are prone to opening against any external or internal forces, resulting in seed shattering. A reduction of the sensitivity to pod opening would not only increase the productivity of the crop but also allow the use of combine harvesters, which would reduce labor costs. An increase in pod shattering resistance is more likely to be associated with differences in the pod wall and dehiscence zone (DZ) development. The DZ comprises three to four layer of cells separating the heavily deposited lignin cells of the pericarp and the replum, which consist of thickened sclerenchyma and vascular cells [9,10]. These vascular traces move from the pod wall to the stalk and the replum. Pod opening takes place when an external force fractures the delicate vascular cells, thereby allowing the valves to separate and seeds to fall on the ground [11]. A central ridge of the replum region divides the two valves. On the replum side of the valve margins, a separation layer forms, which allows detachment of the valve from replum cells. On the valve side, deposition of lignin contents makes a rigid layer of lignified cells continuously along the endocarp-a layer, which is called the endocarp-b layer [12]. Vascular traces situated near the inner edges of the DZ help to secure the valves and make them more difficult to open. Any change in the structure of the DZ is associated with the shattering resistance or susceptibility of the cultivar [10]. In the model plant *Arabidopsis* (*Arabidopsis thaliana*), the establishment of organ identity activates the expression of FILAMENTOUS FLOWER (FIL) and YABBY3 (YAB3) genes [13]. FIL and YAB3 activate the FRUITFULL (FUL) and SHATTERPROOF1/2 (SHP1/2) redundantly with the JAGGED (JAG) gene. FUL and SHP1/2 genes are necessary to form the strips of the valve margin tissues, including lignified and separation layers that allow the fruit to shatter and disperse seeds [12,14]. Several genes are related to pod shattering [7,15,16,17,18,19,20,21,22], carpel [23,24,25], stamen [26,27], and, as a whole, lateral organ development [28,29,30,31,32,33]. JAG gene coding for C_2_H_2_ zinc finger transcription factor functions as a direct mediator between genes that control the identity of tissues, lateral organs, and cellular activities required for organ growth. HANABA TARANU (HAN) and PINHEAD (PNH) are transcription factors that promote each other and biochemically interact to regulate meristem organization. HAN physically interacts with and directly stimulates JAG to regulate floral organ development [34]. JAG function is required to maintain the integrity of boundaries between cell groups with indeterminate or determinate fates.

In this study, we mutagenized five homoeologs of the JAG gene by the CRISPR/Cas9 system in *B. napus* to characterize the zinc finger transcription factor genes that are broadly expressed in all lateral organs. Knockout mutagenesis of all JAG homoeologs produces serrated leaves and unorganized cell identity in floral primordia. Lack of cell identity in the floral organ of *B. napus* generated a number of cells with deformed pods. Among them, we identified a mutant in which only a BnJAG.A08 paralog of the JAG gene was mutated. The BnJAG.A08 mutant produced changes in DZ development, resulting in increased replum area, shortened pod length, and enhanced resistance to pod shattering.

## 2. Materials and Methods

### 2.1. sgRNA Design and Vector Construction

For targeted mutagenesis, JAG homoeologs (BnaA02g13870D, BnaC06g30050D, BnaA07g27150D, BnaC02g18270D, and BnaA08g24290D) were identified from the *B. napus* genome by blasting *Arabidopsis thaliana* genomic sequences, including the AtJAG (At1G68480.1) gene and its paralogous copy NUBBIN (NUB) (At1G13400.1). A phylogenetic tree was constructed by using an online phylogenetic tool (http://www.phylogeny.fr/). According to the phylogeny of JAG homoeologs, a multiplex genome editing tool was used to induce mutagenesis at once. According to the principle of endogenous CRISPR/Cas9 mediated multiplex genome editing, the vector was constructed by using three sgRNAs, as reported previously [35]. The first and second sgRNAs contained a 19 bp target sequence and the third sgRNA contained a 20 nt sequence from the exon region. The CRISPR-P 2.0 online web tool was used for computer-aided design of highly efficient sgRNA with minimal off-target effects [36]. In addition, the specificity of those sgRNAs was confirmed by blasting the rapeseed genome database (http://www.genoscope.cns.fr/blat-server/cgi-bin/colza/webBlat). The expression cassette carried three sgRNAs, enabling us to target all five homoeologs of the JAG gene. The oligo sequences used in constructing the sgRNA vectors are listed in Table S1.

### 2.2. Plant Materials and Vector Transformation

*B. napus* variety “Zhongshuang 6” (ZS6) was used in this experiment, and seeds were collected from the germplasm resource of the Oil Crop Research Institute, Chinese Academy of Agricultural Sciences (OCRI-CAAS), Wuhan, China. The transformation vectors containing three sgRNAs in the expression cassette were introduced in hypocotyls of *B. napus* by the *Agrobacterium tumefaciens* strain (*GV3101*). We followed the same protocol in *B. napus* transformation, as described by Li et al. (2018) [35]. The plants were cultivated in a growth room (16 h/8 h period at 22 °C) and in an experimental station (OCRI-CAAS) in Hanchuan for subsequent T1 generations. Seeds were collected from the T1 generation grown in controlled room conditions (16 h/8 h period at 22 °C) for T2 generation.

### 2.3. DNA Extraction and Identification of Positive Mutants

DNA was extracted from leaf tissues of callus-generated plants by cetyltrimethylammonium bromide (CTAB) extraction buffer. Positive transgenic plants were identified by PCR with NPTII gene-specific primers (Table S2) in a PCR reaction and further analyzed by 10% nondenaturing polyacrylamide gel electrophoresis (PAGE), as described by Li et al. (2018) [35]. After PAGE-based screening (primer details in Table S3), mutants were analyzed by Sanger sequencing of the PCR product (Table S4).

### 2.4. Phenotypic Characterization of Pods

For the undifferentiated cells, the callus was sliced with a sharp stainless-steel blade and the phenotype was observed under a stereo fluorescent microscope (Olympus-SZX16). However, BnJAG.A08 mutants produced pods in place of a callus-like phenotype. For these mutants, the pod length (cm), the number of seeds per pod, pod width (cm), and shattering resistance index (SRI) were evaluated in three biological repeats. Thirty pods were pooled from 10 plants to make one composite sample with three replications to count the number of seeds per pod and measure the pod length and width. A Vernier caliper was used to measure the width and length of the pod. The number of seeds per pod was counted manually for each composite sample. Sixty mature pods were subjected to measurement of the SRI by using a random impact test (RIT), as described previously by Liu et al. (2016) [17].

### 2.5. RNA Isolation and Quantitative Real-Time PCR

Total RNAs were extracted using the RNAprep Pure Plant Kit (TIANGEN, Beijing, China). The plant materials used for RNA extraction were the leaf; stem; and different stages of flower gynoecium, including 15–18. Sample concentrations were quantified by a NanoDrop 2000C spectrophotometer (Thermo Fisher Scientific, Waltham, MA, USA). For cDNA synthesis, a total of 1 µg of RNA for each sample was treated with DNase I to eradicate the genomic DNA and was then used as a template for reverse transcription (QuantiTect Reverse Transcription Kit; Qiagen, Hilden, Germany). The expression level of the ACTIN gene in *B*. *napus* was used to standardize the RNA sample for each RT-PCR. qPCR was performed with SYBER Green Master Mix (Novogene, Beijing, China) using a LightCycler 480 real-time PCR system (Applied Biosystem, Waltham, MA, USA) with three biological replicates and three technical replicates for each sample and ACTIN as the internal control.

### 2.6. Staining of Pod Transverse Section of BnJAG.A08 Mutant

Balanza et al. (2016) found that lignin contents were detected in a vascular bundle at the end of the 17-B stage, so we decided to focus on this late stage of 17-B and the earlier stage of 18, just before the pod turns yellow [37]. Pod stage (17-B) was selected based on the information of valve margin differentiation to identify the lignified and separation layers. Pods of the BnJAG.A08 mutant line (BnJAG-33) were fixed in 50% formalin-aceto-alcohol (FAA) solution overnight. Sample dehydration was done by passing through a series of dehydrating solutions (tertiary butyl alcohol (TBA), 95% ethanol, absolute ethanol, and distilled water). Dehydrated samples were transferred to tubes half filled with TBA and molten paraplast (paraffin wax) for better wax penetration in tissues. After proper orientation of the tissue, the blocks were quickly cooled in a refrigerator. Trimming of wax blocks was performed with a cutter in such a way that all the edges of the block were parallel with plane surfaces. These wax blocks containing embedded tissue were then mounted on square wooden blocks for microtomy. Transverse section of 8–10 μm thickness was prepared by using a microtome and straddled on a slide by using a water bath before dewaxing in histo-clear. Slides were stained with 0.5% Safranin O for 30 min. For lignin-specific staining, pod cross sections were placed in 1% phloroglucinol for 2 min and subsequently acidified with drops of 37% hydrochloric acid. Images of pod cross sections were visualized and imaged under an Olympus IX71 inverted microscope.

### 2.7. Statistical Analysis

The statistical significance of the differences among the means was analyzed by using Duncan’s multiple range test [38] in IBM SPSS statistics (SPSS Inc. IBM, New York, NY, USA).

## 3. Results

### 3.1. Sequence Analysis of BnJAG Gene

**Figure 1:**
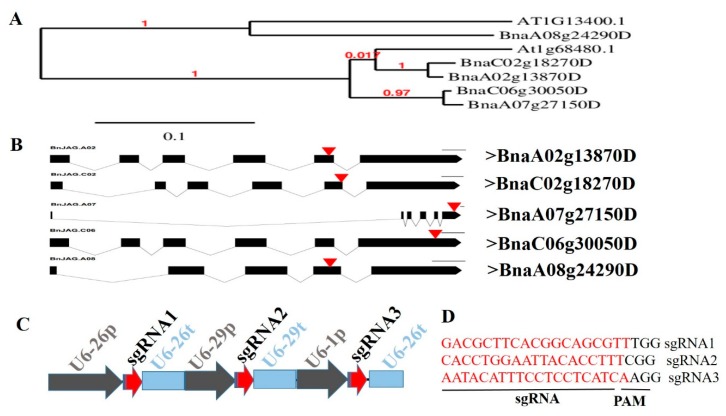
Phylogenetic tree of the JAGGED (JAG) gene and sgRNA expression cassette. (**A**) The evolutionary relationship between the AtJAG sequence and its paralogous copy AtJAG-Like-NUB with *Brassica napus* JAG sequences (>BnaA02g13870D, >BnaC06g30050D, >BnaA07g27150D, >BnaC02g18270D, and >BnaA08g24290D). (**B**) Black boxes indicate the exons and black lines show intron regions. The black box with the red triangle arrow shows the target sites. (**C**) Construction of expression cassettes for multiplex genome editing of five JAG homoeologs with three sgRNAs. (**D**) Three sgRNAs used to construct a vector for the targeted mutagenesis of five homoeologs. By using sgRNA1, BnJAG.C06 and BnJAG.A07 regions were targeted. By using sgRNA2, BnJAG.A02 and BnJAG.C02 homoeologs were targeted. By using sgRNA3, the BnJAG.A08 homoeolog was targeted.

Five BnJAG homoeologs were aligned with *Arabidopsis* genomic sequences to construct a phylogenetic tree among the descendent taxa and evolutionary relationship. Four homoeologs of *B. napus* (BnaA02g13870D, BnaC06g30050D, BnaA07g27150D, and BnaC02g18270D) showed gene divergence at the same speciation event with the AtJAG-1 gene (AT1G68480.1). BnaA08g24290D (BnJAG.A08) showed another speciation event and exhibited lineages with the paralogous copy NUBBIN-like AtJAG gene (AT1G13400.1) (Figure 1A). All homoeologs of the BnJAG gene were targeted with the multiplex genome-editing tool of the CRISPR/Cas9 system by constructing an expression vector with three sgRNAs (Figure 1B–D). By using three sgRNA multiplex tools, the fourth exon of BnaA08g24290D (BnJAG.A08), the fifth exon of BnaA02g13870D (BnJAG.A02) and BnaC02g18270D (BnaJAG.C02), and the sixth exon of BnaC06g30050D (BnJAG.C06) and BnaA07g27150D (BnaJAG.A07) were targeted (Figure 1B).

To avoid off-target effects, blasting analysis was performed on three sgRNA sequences using the rapeseed genome database. The sgRNA sequence blasting results demonstrated that all three sgRNAs can specifically target five BnJAG homoeologs at their genome site (Figure S1).

### 3.2. Knocking Out All Five JAG Homoeologs Dramatically Hampered Pod Development

**Figure 2:**
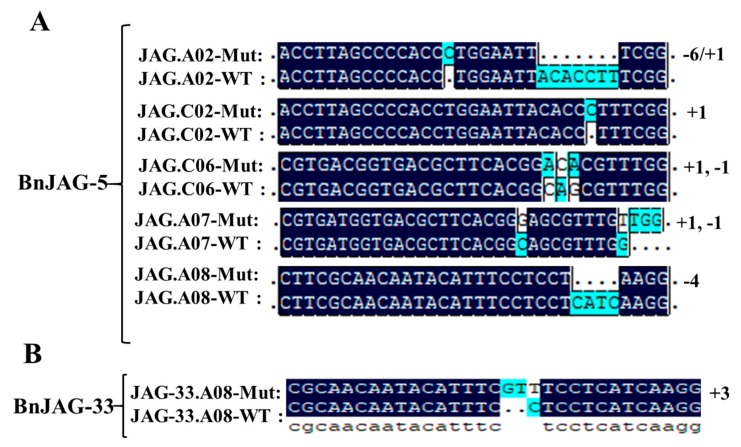
CRISPR/Cas9-induced mutation in JAG homoeologs. (**A**) Mutagenesis in all homoeologs of the JAG gene. Insertion and deletions were identified from 1 to 6 bp. (**B**) Mutagenesis in BnJAG.A08 homoeolog with a 3 bp insertion at the targeted region.

**Figure 3:**
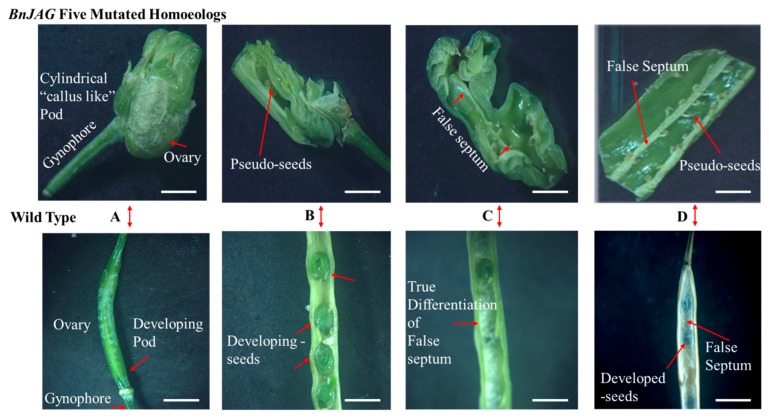
Undifferentiation of cell identity in lateral organs of JAG mutants. (**A**) Mutant exhibited cylindrical hollow callus above the gynophore, while the wild type (WT) showed a normal developing pod. (**B**) Pseudoseeds inside the hollow undifferentiated cells. These seeds did not develop and gain complete size. The WT phenotype showed developing seeds with complete size. (**C**) An incomplete, false septum inside the cylindrical callus; the false septum did not develop in the entire callus, but in the WT pod, the false septum developed entirely along the length of the pod. (**D**) Pseudoseeds are visible on both sides of the dividing layer of the false septum but do not have the potential size to grow for the next generation. WT seeds gained full size, including a developed false septum as a dividing layer of seeds and pod valves. Scale bar corresponds to 1 mm length.

From 106 callus-generated plants, 41 positive transgenic plants were identified by PCR amplification of the NPTII marker gene (Figure S2). After PAGE-mediated screening, 25 plants were identified with at least one mutated homoeolog. Among these 25 mutants, 6 plants were recognized, carrying mutagenesis at all five homoeologs. To verify the PAGE-based screening results, one mutant line (JAG5) was verified by Sanger sequencing (Figure 2A). Sequence alignment results demonstrated that the protein sequences of all five BnJAG homoeologs were changed in mutant line BnJAG-5 (Figure S3). Our previous results suggested that the PAGE-based mutation screening method is a very reliable way to identify CRISPR/Cas9-induced mutation and the effectiveness is over 90% (Li et al., 2018). Thus, only the BnJAG-5 mutant line was further sequenced by Sanger sequencing in the T0 generation. Sequence analysis results suggested that the insertions (ranging from 1 to 4 bp) and deletions (ranging from 1 to 6 bp) were induced at the target sites (Figure 2A). In our amino acid sequence alignment analysis, the protein sequence of all five BnJAG homoeologs was significantly changed in the BnJAG-5 mutant line (Figure S3). Consistent with our PAGE identification data, the phenotype observation results suggested that all six mutated plants exhibited similar phenotypes in pod development (Figure 3). Knocking out all five JAG homoeologs severely affected pod development. The mutants formed a pod with a cylindrical body that developed around the transmitting tract cells, which guide the pollen tube from the stigma to the ovary (Figure 3A). The ovary was comprised of a number of cells, most likely callus, without distinctive differentiation of valves, replum, septum, and valve margins (Figure 3A,B). More likely, this “callus-like pod” was formed from the tip of the gynophore inside of the ovary wall (Figure 3B,C). The cylindrical body developed some pseudoseeds (Figure 3B), but all pseudoseeds were unproductive. Pseudoseeds were divided into halves with a layer of cells (probably false septum) that divided the pseudofruit. However, the false septum always divided the fruit, stretching from one side to the other of the replum along the length of the pod (Figure 3C). However, in the BnJAG-5 mutant line, a number of cells (callus) did not exhibit clear false septum, replum, and vascular structure. The tissue expression pattern of the five BnJAG homoeologs suggested that most of them expressed at floral organs, including petals and stamens, and the early fruit development stage (Figure S6). Thus, BnJAG genes probably play crucial roles in the regulation of pod development.

### 3.3. Mutagenesis in BnJAG.A08 Enhanced Replum Width in the Dehiscence Zone

However, we found that the siliques of the BnJAG-33 mutant line exhibited more resistance to pod shattering when using a palm to press them among those of the T0 mutant lines. Thus, the BnJAG-33 line was selected for sequencing analysis, and the sequencing data suggested that only BnJAG.A08 loci were mutated in the BnJAG-33 line (Figure 2B). Protein sequence alignment results suggested that the amino acid sequence changed from 104 to 109 in the BnJAG-33 line (Figure S4).

**Figure 4:**
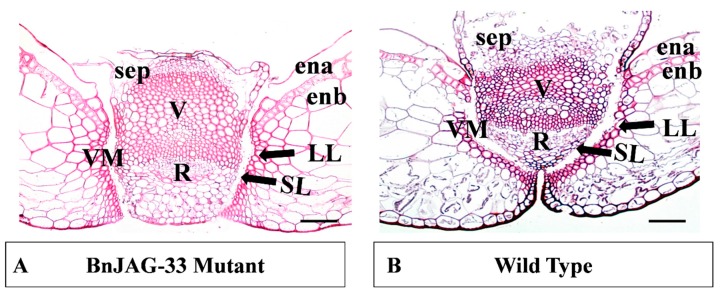
Transverse section of a stained pod of wild type and BnJAG.A08 mutant. (**A**) Phenotype of the BnJAG-33 mutant, in which only the BnJAG.A08 homoeolog was mutated. Replum size was increased in the BnJAG-33 mutant. It showed less deposition of lignin contents in lignified and separation layer development. (**B**) Shorter replum region; fully established separation and lignified layers to generate a dehiscent phenotype. Bar scale is 100 µm. sep—septum; ena—endocarp-a; enb—endocarp-b; V—vascular; VM—valve margins; R—replum; SL—separation layer; LL—lignified layer.

To further confirm the pod shattering resistance phenotype, a pod transverse section experiment was performed on T1 progeny of the BnJAG-33 line. The transverse section results suggested that knockout mutation of the BnJAG.A08 homoeolog caused decisive changes in the DZ by increasing the replum region, as compared with the wild type (WT) (Figure 4). The four other homoeologs were verified multiple times in the T1 generation, which were derived from a single T0 mutant (BnJAG-33). To be safe, we found a transgene-free mutant in the T2 generation. We verified other target sites by Sanger sequencing and found that only the BnJAG.A08 homoeolog was mutated in BnJAG-33 plants (Figure S5 and Figure 2B). After staining the pods, it was observed that BnJAG.A08 mutations influenced the differentiation of lignified and separation layers. A reduced lignification pattern was observed in the DZ of the mutant (Figure 4A). Mutants exhibited an oversized replum with an enlarged width adjacent to the valve margin, as compared with the wild-type plants (Figure 4A,B). In mutants, deposition of lignin contents was observed in the oversized replum. Cells were enlarged in the replum and vascular bundle, which generated fruit with a bumpy shape.

### 3.4. Inheritance Pattern of Mutagenesis at BnJAG.A08 Homoeolog

One construct carrying expression cassettes with three sgRNAs for five targeted genes assured a high mutation rate in all homoeologs. Unfortunately, we did not obtain any seeds for further generations. However, mutagenesis in the BnJAG.A08 homoeolog (BnJAG-33) was inherited by the next generation and generated mutants and wild-type plants with a ratio of 1:1 in the T2 generation. Due to expression cassettes with three sgRNAs, we decided to determine a stable transgene-free genotype with indels at the target site of BnJAG.A08. Two mutants out of 12 T1 plants exhibited insertion at the target site (Table S7 and Figure S5). In the T2 generation, 11 out of 21 plants showed insertion at the targeted region (Table S8).

### 3.5. Variation of Pod Phenotype and SRI

**Figure 5:**
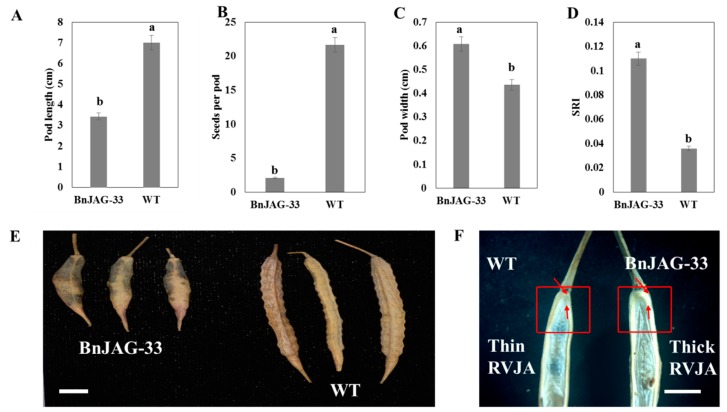
Comparison of replum–valve joint area (RVJA) and phenotypic characteristics of the pod. (**A**) Mutant pod length decreased significantly compared with WT. (**B**) Number of seeds per pod was decreased in mutants significantly compared with WT. (**C**) Pod width was increased in mutants with bumpy shaped fruit, while WT exhibited a slim and straight phenotype. (**D**) SRI was increased ~2-fold in mutants compared with WT. (**E**) Comparison of pod of BnJAG-33 mutant and WT, showing bumpy shape in mutants. Length of the pod in mutants was nearly half that of WT pods. (**F**) A thick RVJA was exhibited in mutants and a thin RVJA in WT, as shown in the red rectangle. Scale bar in (**E**) represents 1 cm. Scale bar in (**F**) represents 5 mm.

Knocking out the BnJAG.A08 homoeolog negatively contributed to the development of pods and greatly affected the agronomic parameters of the fruits. Fruit valves of the mutant (BnJAG-33) were affected by reducing the valve size (3.432 ± 0.17 cm) as compared with the WT plants (7.008 ± 0.36 cm) (Figure 5A). The number of seeds per pod was greatly reduced in mutants (2.11 ± 0.64 cm) compared with WT plants (21.67 ± 1.21 cm) (Figure 5B). Pods showed a continuous outward and inward fashion with twisted valves along the length of the pod (Figure 5E). Mutants exhibited bumpy fruit of widened girth (0.608 ± 0.01 cm) compared with the WT pods (0.436 ± 0.01 cm) (Figure 5C). Analysis of variance indicated that mutants had a highly significant impact on pod length, number of seeds per pod, and pod width. All three traits significantly varied (*p* < 0.001 ***) from wild type to mutant (Table 1). BnJAG-33 mutants showed a ~2-fold higher SRI than WT plants (Table 1, Figure 5D). The increased resistance to shattering may be due to less lignin deposition in the lignified layer (Figure 4A), the bumpy shape of the fruit (Figure 5E), reduction in pod size, and a thick replum–valve joint area (RVJA) (Figure 5F).

### 3.6. Analysis of Transgene-Free Mutants

**Figure 6:**
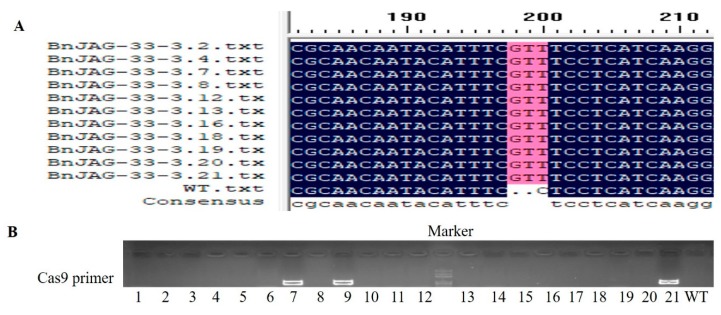
Transgene-free analysis in T2 generation. (**A**) Twenty-one plants were grown from single T1 mutants (BnJAG-33-3), which were categorized from BnJAG-33-3.1 to BnJAG-33-3.21. Among the 21 T2 plants, 11 plants were identified as mutants and the rest exhibited a WT genotype. (**B**) Among the 11 mutants, 9 mutants were identified as transgene-free plants and 2 mutants were carrying the expression cassette. However, BnJAG-33-3.9 were identified with the WT genotype, but it was carrying the expression cassette. Note: 21 T2 descendants (BnJAG-33-3.1 to BnJAG-33-3.21) derived from BnJAG-33-3 were numbered from 1 to 21, as shown below the gel figure.

BnJAG-33 mutants were examined for transgene-free analysis by using Cas9 primers (Figure 6B, Table S5). Among the 21 descendants of the mutants, the PCR product was only detected in three plants, whereas the other 18 plants did not show any band of Cas9 sequences in T2 mutants. Among the 11 mutants that exhibited stable genotypic mutagenesis (Figure 6A), 9 mutants were identified as transgene-free plants (Figure 6B).

### 3.7. Off-Target Prediction

To detect the number of off-target cleavage sites by the CRISPR/Cas9 system, CRISPR-P 2.0 online software was used to predict the off-target loci of sgRNA1–3. We did not find a single guide sequence that carried zero mismatches. All predicted off-target sgRNA sequences were carrying one to four mismatches with a low off-score. To be safe and avoid misleading information, off-target sites (BnaA02g13870D, BnaA07g27150D, BnaC06g30050D, BnaC02g18270D, BnaA05g16310D, and BnaA06g07380D) were analyzed by Sanger sequencing. We did not find a single off-target site in the mutant line BnJAG-33-3 (Figure S7). Our focused line BnJAG-33-3 was double-checked to identify the off-target sites, especially in BnaA05g16310D and BnaA06g07380D (off-target loci of sgRNA3). However, we did not find any off-target mutagenesis at either locus (Figure S7E,F).

## 4. Discussion

The JAG gene is an important growth regulator that encodes a protein with a single C_2_H_2_ zinc finger domain for lateral organs in *Arabidopsis* and the Brassicaceae family. The JAG gene can activate the growth of many tissues, including bracts and lateral organs [28,39,40] and suppresses the premature differentiation of tissues, which is necessary for the formation of distal regions by regulating auxin expression [41,42,43]. Particularly, JAG sculpts floral organs by directly repressing genes that control entry into DNA replication [44]. It functions as a direct mediator between the genes that manage the identity of organs, tissue differentiation, and cellular proliferation activities required for lateral organ growth [45].

Loss of JAG function generates unorganized lateral organs, and the most robust defects are due to a drastic change in gene expression that entails the initiation of lateral plant organs [28,46]. JAG gene silencing in *Aquilegia* resulted in flowers with elongated petals, abnormal stamens, and the absence of carpels [47]. The JAG gene, with its paralog copy NUB [27], is thought to redundantly play a role in generating proliferation in the distal region of lateral organs. Complete loss of JAG homoeologs has been characterized in tomato LYRATE (LYR) and rice OPEN BEAK (OPB) sequences. These mutants exhibited impaired growth of lateral leaflets (compound and simple leaves) and defects in floral organ identity, including the development of mosaic organs, gluminous lodicules, staminoid lodicules, and pistiloid stamens [48,49]. Due to the loss of function of all JAG homoeologs, complete fruit could not develop; instead, a cylindrical callus inside the valves was exhibited with undifferentiated cells in the septum, replum, seeds, and pod valves. Our results, showing significant abnormalities and undifferentiation of cell identity, are consistent with eudicot *Arabidopsis*, in which JAG mutants exhibited narrower and irregularly shaped organs compared with the wild type [33]. In JAG loss-of-function mutants, lateral organs were not developed completely, and there were robust defects in the distal regions and suppressed cell cycle activity in the mutants with irregular margins [28]. Expression of JAG-lateral organ-D (JLO) in transgenic plants of *Arabidopsis* caused seedling lethality organ fusion, confirming that JLO is essentially required to repress the growth of developing organs [42] and a loss of function of JAG and FIL activity exhibited oversized replum in the mutants [46]. We did not obtain any seeds in JAG mutants with all mutated homoeologs. We found some plants that did not contain callus-type cells and that generated some seeds with bumpy-shaped and short pods. In BnJAG.A08 knockout mutants, the pod length decreased significantly, and the replum area increased compared with the wild-type plants (Figure 4A and Figure 5C). Our results are consistent with the results that showed that a loss of function of the AtJAG gene led to enhanced replum size in *Arabidopsis* [46]. In addition, the fruit produced by these mutants as well as in *Arabidopsis* mutants showed a reduction in length and bumpy-shaped lateral organs [50]. An increase in the RVJA enhanced shattering resistance in JAG mutants, whereas WT plants exhibited a thin RVJA, as shown in Figure 5D. Hu et al. (2015) and Chung et al. (2013) revealed that a thick and enlarged replum structure is a main determining factor for higher resistance to pod shattering in rapeseed and *Arabidopsis*, respectively [51,52].

Researchers have achieved an understanding of the interaction of At-JAG1 and AtJAG-Like-NUB genes, which promotes lateral cell proliferation in a sophisticated manner and regulates shattering-related genes. AtJAG1 and AtJAG-Like-NUB genes work redundantly. JAG controls cell proliferation in stamens and carpel development, whereas NUB is expressed in a polar manner on the adaxial side [27,53]. However, we are still in the early stages of determining how these cell identities are fully established in terms of knockout of all JAG homoeologs together and independently to realize the full potential of the sequences.

Our current results, therefore, provide evidence that knockout mutation of all JAG homoeologs leads to undifferentiated cell proliferation in *B. napus* lateral organs, especially pods, which enclose the ovules. Not a single pod bore productive seeds, but pseudoseeds of minute size and shape were developed. Hollow and cylindrical callus-like pods, instead of normal ovary valves and undifferentiated cells, produced pseudoseeds, resulting in no seeds for future generations. However, the BnJAG.A08 mutation generated a widened replum and bumpy, short fruit. A widened replum at the junction of the valves increased pod shattering resistance. From this mutated plant, we generated transgene-free and off-target-free plants in *B. napus* with bumpy-shaped fruit, a widened replum region adjacent to the valve margins, and a short pod length. Knocking out the JAG homoeolog BnJAG.A08 enhanced the replum region, which followed the regulatory mechanism elaborated in *Arabidopsis thaliana*. Based on this phenotype, this gene also was inferred to work upstream of many pod-shattering-related genes, including SHP1/2, IND, and ALC. However, there was no experimental evidence to demonstrate the function of the JAG gene in pod shattering resistance. For the first time, JAG function has been elaborated in pod shattering resistance. Most importantly, we performed this study on the cash crop *B. napus*. Such a study can have a crucial role in understanding the regulatory web of pod shattering resistance.

## Figures and Tables

**Table 1:** Descriptive statistics and analysis of variance for pod phenotypic traits.

Trait	BnJAG-33 (M)		Wild Type (WT)		Variance	Homogeneity	
	Range	Mean	Range	Mean	Treatment ^a^	M ^b^	WT ^c^
Pod length (cm)	3.18–3.71	3.432 ± 0.17	6.42–7.5	7.008 ± 0.36	***	b	a
Seeds per pod	1.1–3.1	2.11 ± 0.64	20.1–23.7	21.67 ± 1.21	***	b	a
Pod width (cm)	0.58–0.63	0.608 ± 0.01	0.41–0.46	0.436 ± 0.01	***	a	b
SRI		0.11		0.036			

^a^ Asterisks show significance level of variance (*** ~ *p* < 0.001). ^b^ M corresponds to a mutant of BnJAG-33 (BnJAG.A08). ^c^ WT corresponds to wildtype. Abbreviation: SRI—shattering resistance index.

## Supplementary Materials

The following are available online at https://www.mdpi.com/2218-273X/9/11/725/s1, Figure S1: sgRNAs blasting from *Brassica napus* genome database, Figure S2: Transgenic identification by using NPTII primers, Figure S3: Predicted protein sequence of BnJAG-5 mutant line, Figure S4: Predicted protein sequence, Figure S5: Mutant identification in T1 generation, Figure S6: BnJAG gene expression in *Brassica napus*, Figure S7: Off target analysis, Table S1: Sequence of three gRNA expression cassettes for *Brassica napus*, Table S2: NPTII primers to identify the positive mutants, Table S3: PCR amplification and Sanger sequencing, Table S4: PAGE-based screening primers, Table S5: Oligo’s for transgene-free analysis in BnJAG-33 mutants, Table S6: T0 generation of JAG mutants, Table S7: T1 generation of JAG mutants, Table S8: T2 generation of JAG mutants, Table S9: Primers of BnJAG homoeologs for qRT-PCR analysis.

## References

[1] Nagaharu U. (1935). Genome analysis in *Brassica carinata* with special reference to the experimental formation of *Brassica napus*, a peculiar mode of fertilization. Jpn. J. Bot..

[2] Chalhoub B., Denoeud F., Liu S., Parkin I.A.P., Tang H., Wang X., Chiquet J., Belcram H., Tong C., Samans B. (2014). Early allopolyploid evolution in the post-neolithic *Brassica napus* oilseed genome. Science.

[3] Friedt W., Snowdon R. (2012). Oilseed rape. Handbook of Plant Breeding.

[4] Prohens J. (2011). Plant breeding: A success story to be continued thanks to the advances in genomics. Front. Plant. Sci..

[5] Dresselhaus T., Hückelhoven R.J. (2018). Biotic and Abiotic Stress Responses in Crop Plants. Agronomy.

[6] Hu Q., Wei H., Yan Y., Zhang X., Liu L., Shi J., Zhao Y., Qin L., Chen C., Wang H. (2017). Rapeseed research and production in China. Crop. J..

[7] Raman H., Raman R., Kilian A., Detering F., Carling J., Coombes N., Diffey S., Kadkol G., Edwards D., Mccully M. (2014). Genome-wide delineation of natural variation for pod shatter resistance in *Brassica napus*. PLoS ONE.

[8] Jie K., Sun Y., Liu T., Zhang P., Zhou M., Wu J., Zhou G. (2016). Physiological mechanisms behind differences in pod shattering resistance in rapeseed (*Brassica napus* L.) Varieties. PLoS ONE.

[9] Child R.D., Summers J.E., Babij J., Farrent J.W., Bruce D.M. (2003). Increased resistance to pod shatter is associated with changes in the vascular structure in pods of a resynthesized *Brassica napus* line. J. Exp. Bot..

[10] Child R.D., Huttly A.K. Anatomical variation in the dehiscence zone of oilseed rape pods and its relevance to pod shatter. Proceedings of the 10th International Rapeseed Congress.

[11] Meakin J.P., Roberts J.A. (1990). Dehiscence of fruit in oilseed rape (*Brassica napus* L.). I. Anatomy of pod dehiscence. J. Exp. Bot..

[12] Dinneny J.R., Weigel D., Yanofsky M.F. (2005). A genetic framework for fruit patterning in *Arabidopsis thaliana*. Development.

[13] Eshed Y., Baum S.F., Bowman J.L. (1999). Distinct mechanisms promote polarity establishment in carpels of *Arabidopsis*. Cell.

[14] Dinneny J.R., Yanofsky M.F. (2005). Drawing lines and borders: How the dehiscent fruit of *Arabidopsis* is patterned. Bioessays.

[15] Tao Z., Huang Y., Zhang L., Wang X., Liu G., Wang H. (2017). BnLATE, a Cys2/His2-type zinc-finger protein, enhances silique shattering resistance by negatively regulating lignin accumulation in the silique walls of *Brassica napus*. PLoS ONE.

[16] Kord H., Shakib A.M., Daneshvar M.H., Azadi P., Bayat V., Mashayekhi M., Zarea M., Seifi A., Ahmadraji M. (2015). RNAi-mediated down-regulation of *SHATTERPROOF* gene in transgenic oilseed rape. 3 Biotech.

[17] Liu J., Wang J., Wang H., Wang W., Zhou R., Mei D., Cheng H., Yang J., Raman H., Hu Q. (2016). Multigenic control of pod shattering resistance in chinese rapeseed germplasm revealed by genome-wide association and linkage analyses. Front. Plant Sci..

[18] Peng P.F., Li Y.C., Mei D.S., Colasanti J., Fu L., Liu J., Chen Y.F., Hu Q. (2014). Expression divergence of *FRUITFULL* homeologs enhanced pod shatter resistance in *Brassica napus*. Genet. Mol. Res..

[19] Ogutcen E., Pandey A., Khan M.K., Marques E., Penmetsa R.V., Kahraman A., Von-Wettberg E.J. (2018). Pod Shattering: A homologous series of variation underlying domestication and an avenue for crop improvement. Agronomy.

[20] Zhai Y., Cai S., Hu L., Yang Y., Amoo O., Fan C., Zhou Y. (2019). CRISPR/Cas9-mediated genome editing reveals differences in the contribution of *INDEHISCENT* homologues to pod shatter resistance in *Brassica napus* L.. Theor. Appl. Genet..

[21] Stephenson P., Stacey N., Brüser M., Pullen N., Ilyas M., O’Neill C., Wells R., Østergaard L. (2019). The power of model-to-crop translation illustrated by reducing seed loss from pod shatter in oilseed rape. Plant Reprod..

[22] He H., Bai M., Tong P., Hu Y., Wu H. (2018). CELLULASE 6 and MANNANASE 7 affect cell differentiation and silique dehiscence. Plant Physiol..

[23] Groszmann M., Paicu T., Alvarez J.P., Swain S.M., Smyth D.R. (2011). SPATULA and ALCATRAZ, are partially redundant, functionally diverging bHLH genes required for *Arabidopsis* gynoecium and fruit development. Plant J..

[24] Kerstetter R.A., Bollman K., Taylor R.A., Bomblies K., Poethig R.S. (2001). KANADI regulates organ polarity in *Arabidopsis*. Nature.

[25] Alvarez J., Smyth D.R. (2002). CRABS CLAW and SPATULA genes regulate growth and pattern formation during gynoecium development in *Arabidopsis thaliana*. Int. J. Plant Sci..

[26] Zhang Y., Wang X., Zhang W., Yu F., Tian J., Li D., Guo A. (2011). Functional analysis of the two brassica ap3 genes involved in apetalous and stamen carpelloid phenotypes. PLoS ONE.

[27] Dinneny J.R., Weigel D., Yanofsky M.F. (2006). NUBBIN and JAGGED define stamen and carpel shape in *Arabidopsis*. Development.

[28] Dinneny J.R., Yadegari R., Fischer R.L., Yanofsky M.F., Weigel D. (2004). The role of JAGGED in shaping lateral organs. Development.

[29] Hepworth S.R., Zhang Y., McKim S., Li X., Haughn G.W. (2005). BLADE-ON-PETIOLE-dependent signaling controls leaf and floral patterning in arabidopsis. Plant Cell.

[30] Ha C.M., Kim G.T., Jun J.H., Soh M.S., Ueno Y., Machida Y., Tsukaya H., Nam H.G. (2003). The BLADE-ON-PETIOLE 1 gene controls leaf pattern formation through the modulation of meristematic activity in *Arabidopsis*. Development.

[31] Nath U., Brian C.W.C., Rosemary C., Enrico C. (2003). Genetic control of surface curvature. Science.

[32] Palatnik J.F., Allen E., Wu X., Schommer C., Schwab R., Carrington J.C., Weigel D. (2003). Control of leaf morphogenesis by microRNAs. Nature.

[33] Ohno C.K., Reddy G.V., Heisler M.G., Meyerowitz E.M. (2004). The *Arabidopsis* JAGGED gene encodes a zinc finger protein that promotes leaf tissue development. Development.

[34] Ding L., Yan S., Jiang L., Zhao W., Ning K., Zhao J., Liu X., Zhang J., Wang Q., Zhang X. (2015). HANABA TARANU (HAN) bridges meristem and organ primordia boundaries through PINHEAD, JAGGED, BLADE-ON-PETIOLE2 and CYTOKININ OXIDASE 3 during flower development in arabidopsis. PLoS Genet..

[35] Li C., Hao M., Wang W., Wang H., Chen F., Chu W., Zhang B., Mei D., Cheng H., Hu Q. (2018). An efficient CRISPR/Cas9 platform for rapidly generating simultaneous mutagenesis of multiple gene homoeologs in allotetraploid oilseed rape. Front. Plant Sci..

[36] Hao L., Ding Y., Zhou Y., Jin W., Xie K., Cheng L. (2017). CRISPR-P 2.0: An improved CRISPR/Cas9 tool for genome editing in plants. Mol. Plant.

[37] Balanzã V., Roig-Villanova I., Di M.M., Masiero S., Colombo L. (2016). Seed abscission and fruit dehiscence required for seed dispersal rely on similar genetic networks. Development.

[38] Duncan D.B. (1955). Multiple Range and Multiple F Tests. Biometrics.

[39] Sauret-Güeto S., Schiessl K., Bangham A., Sablowski R., Coen E. (2013). JAGGED controls arabidopsis petal growth and shape by interacting with a divergent polarity field. PLoS Biol..

[40] Siegfried K.R., Eshed Y., Baum S.F., Otsuga D., Drews G.N., Bowman J.L. (1999). Members of the YABBY gene family specify abaxial cell fate in *Arabidopsis*. Development.

[41] Lampugnani E.R., Kilinc A., Smyth D.R. (2013). Auxin controls petal initiation in *Arabidopsis*. Development.

[42] Borghi L., Bureau M., Simon R. (2007). *Arabidopsis* JAGGED LATERAL ORGANS is expressed in boundaries and coordinates knox and pin activity. Plant Cell.

[43] Rolland-Lagan A.-G., Prusinkiewicz P. (2010). Reviewing models of auxin canalization in the context of leaf vein pattern formation in *Arabidopsis*. Plant J..

[44] Schiessl K., Muino J.M., Sablowski R. (2014). *Arabidopsis* JAGGED links floral organ patterning to tissue growth by repressing Kip-related cell cycle inhibitors. Proc. Natl. Acad. Sci. USA.

[45] Donnelly P.M., Bonetta D., Tsukaya H., Dengler R.E., Dengler N.G. (1999). Cell cycling and cell enlargement in developing leaves of arabidopsis. Dev. Biol..

[46] González-Reig S., Ripoll J.J., Vera A., Yanofsky M.F., Martínez-Laborda A. (2012). Antagonistic gene activities determine the formation of pattern elements along the mediolateral axis of the arabidopsis fruit. PLoS Genet..

[47] Min Y., Kramer E.M. (2017). The Aquilegia JAGGED homolog promotes proliferation of adaxial cell types in both leaves and stems. New Phytol..

[48] Horigome A., Nagasawa N., Ikeda K., Ito M., Itoh J.I., Nagato Y. (2010). Rice OPEN BEAK is a negative regulator of class 1 knox genes and a positive regulator of class B floral homeotic gene. Plant J..

[49] David-Schwartz R., Koenig D., Sinha N.R. (2009). LYRATE is a key regulator of leaflet initiation and lamina outgrowth in tomato. Plant Cell.

[50] Xu B., Li Z., Zhu Y., Wang H., Ma H., Dong A., Huang H. (2008). *Arabidopsis* genes AS1, AS2, and JAG negatively regulate boundary-specifying genes to promote sepal and petal development. Plant Physiol..

[51] Hu Z., Yang H., Zhang L., Wang X., Liu G., Wang H., Hua W. (2015). A large replum-valve joint area is associated with increased resistance to pod shattering in rapeseed. J. Plant Res..

[52] Chung K.S., Lee J.H., Lee J.S., Ahn J.H. (2013). Fruit indehiscence caused by enhanced expression of NO TRANSMITTING TRACT in *Arabidopsis thaliana*. Mol. Cells.

[53] Avino M., Kramer E.M., Donohue K., Hammel A.J., Hall J.C. (2012). Understanding the basis of a novel fruit type in Brassicaceae: Conservation and deviation in expression patterns of six genes. EvoDevo.

